# Prevalence of depression and its associated factors among patients with diabetes mellitus at Tirunesh-Beijing general hospital, Addis Ababa, Ethiopia

**DOI:** 10.1186/s12889-020-8360-2

**Published:** 2020-02-22

**Authors:** Nigus Alemnew Engidaw, Abate Dargie Wubetu, Elyas Admasu Basha

**Affiliations:** College of Health Sciences, Debre Berhan University, Po. Box 445, Debre Berhan, Ethiopia

**Keywords:** Prevalence, Depression, Diabetic mellitus, Ethiopia

## Abstract

**Background:**

Depression is one of the common and overwhelming mental disorder in diabetic patients. A little is known about the prevalence and associated factors of depression among diabetic patients at general hospitals. Therefore, this study aimed to assess the prevalence and associated factors of depression among patients with diabetes mellitus at Tirunesh-Beijing General Hospital, Addis Ababa, Ethiopia.

**Methods:**

An institution-based cross-sectional study was conducted from February 8 to April 8, 2019. A systematic random sampling technique was used to select 403 participants. Depression was assessed by using patient health questionnaire-9 (PHQ-9). Oslo 3 social support scale was used to assess social support. The data were entered into Epidata version 3.1 and analyzed by the statistical package for social science version 23 software. We computed bivariate and multivariate binary logistic regressions to assess factors associated with depression. Statistical significance was declared at *P*-value < 0.05.

**Results:**

A total of 403 study participants were interviewed with a response rate of 99%. The prevalence of depression among diabetic patients was 21.3%. In the final multivariate analysis, diagnosed with type II diabetes mellitus, being physically disabled and having poor social support were independent predictors of depression.

**Conclusions:**

Type II diabetes mellitus, poor social support and physical disability were factors associated with depression. An early depression-focused regular screening for diabetic patient should be carried out by trained health professionals. Linkage with mental health service providers also needs to be considered. Clinicians needs to give emphasis to diabetic patients with physically disable and poor social support.

## Background

Diabetes mellitus (DM) is a chronic metabolic condition that occurs when the body cannot use insulin. It is classified as type 1 and type 2 diabetes mellitus [1]. The onset of type 1 and 2 diabetes mellitus is usually begin in childhood and adulthood respectively [2]. It is believed that type 1 diabetes mellitus is caused by genetics, but environmental factors like viruses might trigger the disease. Type 2 diabetes mellitus is caused by several reasons including lifestyle factors and genes [3].

The prevalence of DM is increasing across the world, with 438 million people expected to have the disease by 2030. Over 70% of morbidity and 88% of mortality because of diabetes occur in low and middle-income countries [4, 5]. Because of the differences in the study period and area, the frequency of diabetes mellitus in Ethiopia was ranging from 0.34 to 5.0% [6]. World Health Organization (WHO) estimated that the number of patients with diabetes in Ethiopia is expected to be 1.8 million by 2030. The changes in lifestyle have led to the emerge of non-communicable chronic diseases [6, 7]. The reason of increasing diabetes mellitus in Ethiopia is thought to be limited medical supplies and shortage of skilled human resource. In addition, the major focus of the country is on combating infectious diseases [8].

Being newly diagnosed with diabetes is a major life stress for the patients. Many of them go through the typical stages of mourning such as denial, anger, depression, and acceptance [9]. Depressive disorder is one of the common and overwhelming psychiatric disorder among people with diabetes mellitus [10]. Studies showed that depression is a common comorbid condition in patients with diabetes [11, 12]. The prevalence of depression among patients with DM ranges from 8 to 15%, compared with an estimate of only 3 to 4% in the general population [13, 14]. In Ethiopia, the prevalence of depression among DM patients were ranging from 13 to 40.4% because of the difference in study period, setting and assessment tools [15–17].

The presence of depression in patients with diabetes mellitus is associated with the burden of complications, financial stress, poor overall health status, knowledge of diabetes and poor glycemic control. It also worsens the prognosis of diabetes, increases the noncompliance to the medical treatment, decreases the quality of life, prolong the recovery from diabetes and increases mortality [15, 18–20]. Depression is also a major risk factor in hospital admissions and diabetes-related complications [12].

In Ethiopia, the prevalence and associated factors were varying from place to place. There is little concern about the psychiatric aspects of chronic medical conditions such as diabetes mellitus in general hospitals. Most of the previous researches regarding the prevalence and associated factors of depression in diabetes patients were undertaken at specialized diabetic care settings. Therefore, the aim of this study was to assess the prevalence of depression and associated factors among patients with diabetes mellitus at Tirunesh Beijing General Hospital (TBGH).

The result of the study would help to design more effective programs in the management of comorbid diabetes and depression. Also, the finding of the study will be used as a baseline for other researchers who want to conduct a large scale study in Ethiopia. Those who might be aimed to inquiring the relationship between depression and DM is also benefited from this study. This research finding might help the healthcare providers to initiate early screening, diagnosing and management of depression in diabetic patients.

## Methods

### Study area

This study was conducted at an outpatient department of TBGH, Addis Ababa, Ethiopia. The hospital is fully organized and launched in 2011 with a total number of 376 employees. Now a days, it is estimated to give service for about 605,266 peoples per year. Patients with DM have gotten the treatment in the chronic disease case team of the medical ward. The hospital has also given the psychiatric service at the outpatient level.

### Study design and period

This was an institution-based cross-sectional study conducted from February 8 to April 8, 2019.

### Source and study population

All diabetic patients who were on follow up at the outpatient department were the source population. All patients with DM who came to follow up during the data collection period were considered as the study population.

### Inclusion and exclusion criteria

All diabetic patients aged≥18 years and communicating independently were included. Those who were taking antidepressant drugs for their depressive symptoms were excluded because antidepressant drugs can mask depression signs and symptoms. DM patients who were newly diagnosed at the time of data collection were not part of the study because in a newly diagnosed patient, adjustment disorder is more common than a full-blown depressive symptom [21]. Finally, DM patients who were seriously ill were excluded from this study.

### Study variables

The dependent variable was depression. Independent variables included sociodemographic factors (age, sex, marital status, ethnicity, religion, educational and occupational status), clinical factors (type of DM, FBG level, duration of DM, type of treatment), and psychosocial factors (social support).

### Sample size determination

Single population proportion formula was used to estimate the minimum numbers of samples required for this study. The sample size was calculated with the assumptions of 40.4% prevalence of depression from studies conducted in Felege-Hiwot referral hospital, Bahir Dar [17], 0.4 P, 1.96 Z (standard normal distribution), 95% CI, ⍺ = 0.05, and a 10% non-response rate. Accordingly, a representative sample was calculated to be 407.

### Sampling technique and procedure

Participants were selected for interviews using the systematic random sampling technique. Before the data collection, the total number of diabetic patients who visited the hospital in 2018 was taken from patients’ record. Then the average number of diabetic patients over 2 month’s period was calculated and found to be 930. The sampling interval(k) was determined by dividing the expected number of diabetic patients expected to have a follow-up visit at the time of the data collection to the calculated sample size (K = 930/407 ≈ 2). Finally, eligible individuals were interviewed for every 2 intervals based on the order of their clinical evaluation at the outpatient department until the required sample size was reaching.

### Method of data collection and tools

Data were collected by face-to-face interviews using a pretested semi-structured questionnaire consisting of socio-demographic factors, clinical characteristics, Oslo 3 item social support scale and patient health questionnaire-9 (PHQ-9). The questionnaire of socio-demographic and clinical related information was assessed by using questionnaires adapted from reviewing similar related articles and the patients’ medical record.

The outcome variable (depression) was measured by the nine items of the PHQ-9 tool, validated in east Africa including Ethiopia with a sensitivity and specificity 86 and 67% respectively [22]. It consists of nine items on a four-point Likert scale and scores each of nine DSM-IV criteria for depression [23]. Patients were expected to recall the depressive symptoms which were happening within 2 weeks period and the responses range from 0- (not) to 3- (nearly every day). PHQ-9 score ≥ 5 was considered having depression. The level of social support was assessed by using the Oslo 3 social support scale by asking the patients to rate the level of support they received from family and friends. It is validated in different African countries. The scale ranged from 3 to 14. Poor, intermediate and strong social support was considered for participants who scored 3–8, 9–11 and 12–14 out of 14 respectively [24].

### Data quality assurance

The questionnaire was translated from English to Amharic and back translation to English was done to check its consistency. Before the actual data collection, the Amharic version of the questionnaire was pretested on 5% of the total study participants. Based on the pretest result, a minor modification was done regarding the contents of the questionnaire. Data collectors were trained on the content of the questionnaire and data collection procedures.

### Data processing and analysis

All collected data were checked for completeness and consistency and entered into Epi-data version 3.1 and then exported to SPSS version 23 software for analysis. Descriptive statistics (frequencies, tables, percentages, and means) were computed to explain the socio-demographic characteristics, clinical variables, and depression. Bivariate and multivariate logistic regression analyses were done. Variables that have *p*-value < 0. 20 in the bivariate model were entered into the multivariate analysis to avoid potential confounders. In the multivariate model, variables with *P*-values of less than 0.05 were considered as statistical predictors of depression. The odds ratio with a 95% confidence interval was used to measure the strength of the association.

## Results

### Socio-demographic characteristics

A total of 403 participants took part with a response rate of 99%. The mean age of respondents were 46.4 (±SD = 13.3) years. About 52.4% of the participants were males and a higher percentage (72.7%) of the participants were orthodox in religion. Sixty-two percent of the respondents were married and 21.3% of the participants attended secondary school. Concerning their occupation, 25.6% of the participants were civil servants. The largest proportion (77.9%) of the respondents had an income of ≥1653 Ethiopian Birr (ETB) per month or 56 USA dollars (Table 1).

**Table 1:** Socio-demographic characteristics of patients with diabetes mellitus attending outpatient department at Tirunesh Bejing General Hospital, Addis Ababa, Ethiopia, 2019 (*n* = 403)

Variable	Category	Frequency	Percent
Sex	Male	211	52.4
	Female	192	47.6
Age	18–34	106	26.3
	35–44	80	19.9
	45–54	83	20.6
	≥55	134	33.3
Religion	Orthodox	293	72.7
	Muslim	98	24.3
	Protestant	12	3.0
Ethnicity	Oromo	201	49.9
	Amhara	126	31.3
	Tigre	22	5.5
	Other^a^	54	13.4
Residence	Urban	346	85.9
	Rural	57	14.1
Marital status	Single	70	17.4
	Married	250	62.0
	Divorced	40	9.9
	Widowed	30	7.4
	Separated	13	3.2
Educational status	Can’t read & write	33	8.2
	Able to Read & write only	57	14.1
	Primary school	71	17.6
	Secondary school	86	21.3
	Diploma	73	18.1
	Degree and above	83	20.6
Occupation	Farmer	34	8.4
	Civil servant	104	25.8
	Private employed	82	20.3
	Merchant	83	20.6
	Retire	44	10.9
	Other^b^	56	13.9

^a^ = wolayita, Gurage, Afar ^b^ = Daily laborer, Housewife, student

### Clinical characteristics

From the total interviewee, nearly two-thirds (67.2%) and 32.8% of the participants had type II and type I DM respectively. One third (33.3%) of the respondent was diagnosed at the age of 35–44. The majority (68.5%) of them had the disease for ≤8 years. From those who have been taking medication, 58.1% were on oral hypoglycemic regimen. Over one third (34.7%) of the participants had fasting blood glucose (FBG) level of 101-125 mg/dl and 15.9% of the diabetic patient had diabetic retinopathy (Table 2).

**Table 2:** Clinical and psychosocial characteristics of patients with diabetes mellitus attending outpatient department at Tirunesh Bejing General Hospital, Addis Ababa, Ethiopia, 2019 (*n* = 403)

Variable	Category	Frequency	Percent
Type of DM	Type-I	132	32.8
	Type-II	271	67.2
Age at diagnosis	≤17	18	4.5
	18–34	134	33.3
	35–44	103	25.6
	45–54	89	22.1
	≥55	59	14.6
Duration of DM (in year)	≤8	276	68.5
	9–16	90	22.3
	≥17	37	9.2
DM Rx regime	Insulin	136	33.7
	Oral hypoglycemic	234	58.1
	Insulin plus oral	33	8.2
Duration of DM Rx (in year)	≤8	284	70.5
	9–16	83	20.6
	≥17	36	8.9
No. of prescribed medication administered per day	≤3	377	93.5
	≥4	26	6.5
compliant with medication’	Yes	361	89.6
	No	42	10.4
Reasons for non-compliance (*n* = 42)	No money to buy it	7	1.7
	Drug side effect	13	3.2
	Others	22	5.5
Ever Measured FBG	Yes	342	84.9
	No	61	15.1
FBG level(in mg/dl)	≤100	71	17.6
	101–125	137	34
	≥126	134	33.3
DM complication	Yes	133	33
	No	270	67
Physical disability	Yes	13	3.2
	No	376	93.3
Social Support	Poor	117	29
	Intermidate	127	31.5
	Strong	159	39.5

Abbreviations: *DM* Diabetes Mellitus, *DM RX* Diabetes Mellitus Treatment, *FBG* Fasting Blood Glucose

### Prevalence of depression among diabetes mellitus patients

The prevalence of depression among diabetic patients was found to be 21.3% (95% CI: 16.9, 25.3). From 403 participants; 12.2, 7.4, 4.7, 1.2% have fulfilled the criteria for a mild, moderate, moderately severe and severe form of depression respectively.

### Factors associated with depression among diabetes mellitus patients

In this study, residing in the rural area, diagnosed with type II DM, having fast blood glucose (FBG) level ≥ 126, being physically disabled and having poor social support were associated with depression in the bivariate analysis (Table 3). However, in the multivariate analysis, type II DM (AOR: 2.60, 95%CI =1.26, 5.37), being physically disabled (AOR: 4.70, 95%CI = 1.28, 17.17 and having poor social support (AOR: 4.70, 95%CI = 1.28, 17.17) were found to be the independent predictors of depression among diabetic patients (Table 4).

**Table 3:** Bivariate analysis of depression among patients with diabetes mellitus attending outpatient department at Tirunesh Bejing General Hospital, Addis Ababa, Ethiopia, 2019 (*n* = 403)

Variables	Depression(n)		COR (95% CI)	*P*-value
	Yes	No		
Residence				
Urban^a^	78	268	1.00	
Rural	8	49	0.56(0.25, 1.23)	0.13
Type of DM				
Type I^a^	14	106	1.00	
Type II	72	211	2.58(1.39, 4.79)	0.024
FBG level				
≤100^a^	8	63	1.00	
101–125	22	115	1.507(0.63, 3.58)	0.11
≥126	38	96	3.11(1.36, 7.12)	0.15
Social support				
Poor	45	72	4.34(2.38, 7.9)	0.014
Intermediate	21	106	1.37(0.71, 2.67)	0.032
Strong^a^	20	139	1.00	
Physical disability				
Yes	6	7	3.38(1.10, 10.36)	0.016
No^a^	76	300	1.00	

Abbreviations: *DM* Diabetes Mellitus, *DM RX* Diabetes Mellitus Treatment, *FBG* Fast Blood Glucose, *COR* Crude Odd Ratio, *CI* Confidence Interval

Note:^a^ indicates the reference variables

**Table 4:** Multivariate analysis of depression among patients with diabetes mellitus attending outpatient department at Tirunesh Bejing General Hospital, Addis Ababa, Ethiopia, 2019 (*n* = 403)

Variables	Depression(n)		AOR (95% CI)	*P*-value
	Yes	No		
Residence				
Urban^a^	78	268	1.00	
Rural	8	49	0.43(0.13, 1.39)	0.71
Type of DM				
Type I^a^	14	106	1.00	
Type II	72	211	**2.60(1.26, 5.37)**	**0.021**
FBG level				
≤100^a^	8	63	1.00	
101–125	22	115	1.308(0.53, 3.19)	0.64
≥126	38	96	2.01(0.83, 4.82)	0.67
Social support				
Poor	45	72	**3.61(1.76, 7.36)**	**0.034**
Intermediate	21	106	1.42(0.66, 3.08)	0.81
Strong^a^	20	139	1.00	
Physical disability				
Yes	6	7	**4.70(1.28, 17.17)**	**0.032**
No^a^	76	300	1.00	

Abbreviations: *DM* Diabetes Mellitus, *DM RX* Diabetes Mellitus Treatment, *FBG* Fast Blood Glucose, *AOR* Adjusted Odd Ratio, *CI* Confidence Interval

Note:^a^ indicates the reference variables

## Discussion

The aim of this study was to assess the prevalence and associated factors of depression among people with diabetes mellitus at the outpatient department of TBGH. This study showed that the prevalence of depression was found to be 21.3%. The finding of the current study was in line with a cross-sectional study done in the USA (18%) [11].

The present study was lower than a cross-sectional study conducted in India, Islamic Republic of Iran, Egypt, and Bahrain, 43.4, 74.4, 33.3, 35%, respectively [25–28]. The finding of this study was also lower than the result reported in Ethiopia 40.4 and 43.6% in Bahir Dar and Jimma, respectively [17, 20]. The discrepancy might be due to different assessment tools, health service delivery systems, educational status, lifestyle, and social interaction. For example in Egypt, the MADRS screening tool was used for assessing depression. In the study of Iran and Ethiopia (Felege-Hiwot referral hospital and Jimma University Specialized Hospital), Beck depression inventory (BDII) was used to assess depression. The other reason for the difference might be due to different study settings and participants. For example, the study in Egypt was done among individuals with Type 2 DM.

On the contrary, this study was relatively higher than studies conducted in Malaysia, University of Gondar diabetic clinic and Black-Lion Specialized Hospital, 12.3, 15.4, 13%, respectively [15, 16, 29]. One of the reasons for the difference might because of the different health care settings and likely due to the cut-off score for PHQ-9 being 5. Besides, all of the above studies were conducted on specialized hospitals. Those who are treated in a specialized hospital may have better and comprehensive care because of adequate skilled human power.

The second objective of this study was to identify the associated factors of depression among patients with diabetes mellitus. Accordingly, Patients with Type II diabetes have 2.6 times the odds of having depression than those with Type 1 diabetes mellitus. It is supported by a study conducted in Jimma, Ethiopia [20]. The possible reason might be due to the fact that type II diabetes mellitus has early onset [13].

The odds of developing depression among diabetic patients who had poor social support were 3.61 times more likely when compared with clients who had strong social support. This finding was similar to the study conducted in Ethiopia at Felege-Hiwot referral and Black Lion Specialized Hospitals [17, 19]. This might be due to the fact that social isolation reduces social support, which can have undesirable influence on physical and mental well-being. Having poor social support may leads to delayed diabetic treatment. If the treatment is delayed, the patient will have an early signs of diabetic-related complication which predispose the patient to different psychiatric disorders including depression [30].

Diabetic patients who were physically disabled were 4.7 times more likely to have depression than non-disabled patients. The reason might be due to the fact that physical disability leads to unemployment, less chance to get educational opportunities and social relationships which may predispose the patient to develop depression. The other possible reason is that those who are physical disability may not have adequate physical exercise [31].

### Strength and limitations of the study

The use of a relatively high sample size with a high response rate and using validated tools were the strength of this study. The current study has also some important limitations that should be kept in mind when interpreting the results. This study was conducted in health facilities; hence the findings might not adequately reflect the depression of the entire diabetic patient in the community. The cross-sectional nature of the study design does not confirm a definitive cause and effect relationship.

## Conclusion

Being diagnosed with type II DM, not having good support from family and the society and being physical disabled were the factors associated with depression. Clinicians needs to give emphasis to diabetic patients with physically disable and poor social support. Early detection of depressive symptoms and treating them as a routine component of diabetes care are recommended for clinicians who work closely with diabetic patients.

## Data Availability

The datasets generated and/or analyzed during the current study are not publicly available due to some privacy reasons, but part of the row datasets will be available in the recommended publicly available data repository of BMC or from the corresponding author on reasonable request.

## Abbreviations

AOR: Adjusted odd ratio
CI: Confidence interval
DM: Diabetes mellitus
OPD: Outpatient Department
PHQ-9: Patient Health Questioner nine
USA: United States of America
